# HIV prevalence, sexual and HIV testing behaviors among men who have sex with men in the Republic of Cyprus: 2011-2012 data from a cross-sectional study

**DOI:** 10.1186/1471-2334-14-432

**Published:** 2014-08-06

**Authors:** Magdalini Pylli, Nicos Middleton, Andreas Charalambous, Vasilios Raftopoulos

**Affiliations:** Hellenic Centre for Diseases Control and Prevention, HIV & STIs Office, Athens, Greece; Nursing Department, Cyprus University of Technology, Limassol, Cyprus; Cyprus University of Technology, Nursing Department, Mediterranean Research Centre for Public Health and Quality of Care, 15, Vragadinou Str, 3041 Limassol, Cyprus

**Keywords:** Men who have sex with men, HIV prevalence, Time location sampling, Risk behaviors, Unprotected anal intercourse

## Abstract

**Background:**

The Republic of Cyprus is recognized as a low level HIV epidemic country with strong evidence of an increase in the transmission through the male to male sexual contact. Little is known about the factors that influence the sexual and HIV testing behavior in the Republic of Cyprus.

**Methods:**

This is the first bio-behavioral study among men who have sex with men (MSM) in three major cities in the Republic of Cyprus, conducted between 2011 and 2012. Eligible participants were sampled in gay venues by using time-location sampling.

**Results:**

Estimated HIV prevalence was 2.5%. The mean age of the sample was 29 ± 6.6 years old. One out of three MSM has not been tested for HIV in the last year. Multivariate logistic regression analysis revealed that the educational level (AOR 0.23, 95% CI 0.09-0.55), the cocaine use (AOR 3.78, 95% CI 1.21-11.83) as well as the type of sexual partner i.e. steady vs casual (AOR 0.18, 95% 0.08-0.45) were significantly associated with condom use in the last anal intercourse.

**Conclusions:**

HIV prevalence among MSM in the Republic of Cyprus remains low; however more efforts are needed in order to increase HIV awareness and prevent the expansion of HIV epidemic in broader community.

## Background

HIV epidemic still remains a major public health issue worldwide. Male-to-male sexual contact continues to play an important role in the spread of HIV infection in the United States [1], Canada [2] as well as the European Union and the European Economic Area (EU/EEA) [3]. Specifically in 2012, in EU/EEA countries almost 40% of new infections were attributed to male-to-male sexual contact. HIV transmission among Men who have sex with Men (MSM) accounted for more than half of the HIV cases in nine countries, including Republic of Cyprus [3].

According to the National Statistical Service of the Republic of Cyprus, at the end of 2011 the total number of the inhabitants in the Government controlled area of the Republic of Cyprus was around 862,000, of whom 48.6% were males [4]. The HIV surveillance system in the Republic relies on case reporting via mandatory notification reporting system. By October 2013, the cumulative number of reported HIV positives was 793. Among them 423 were Greek Cypriots (G/C), 2 were Turkish Cypriots (T/C) while 370 were not Cypriots. Of all the cases, 69% were men. Particularly, by October 2013, 46 new cases had been diagnosed. Amongst them, 33 were G/C. The predominant way of transmission among men was male-to-male sexual contact (52%). Sixty six percent of the cases were between 20-39 years of age while 30% were older (40-65 years of age) and 4% were between 0-19 years of age [5]. The Republic of Cyprus has not yet established a second generation surveillance system for HIV [6, 7].

According to a recent review, unprotected anal intercourse (UAI) with an HIV unknown partner, biological factors, co-infections and behaviors associated with anal sex (i.e receptive and insertive anal sex), structural factors, alcohol and illicit drug use as well as network level factors are reported as the main factors that contribute to the HIV epidemic expansion among MSM [8]. Moreover, other psychosocial factors, such as internalised homophobia [9], sexual stigma and discrimination [10], as well as low self-esteem [11, 12] have been implicated in the adoption of risky sexual behaviors among MSM.

Evidence regarding the acquisition of risky behaviors among MSM in each country is important in order to prevent the spread of the disease since the quantification of these behaviors is important for the prevention and for the design of targeted interventions in the future. Several bio behavioral surveys using different recruitment methods have been conducted among MSM in Central and Eastern Europe in order to explore and understand HIV transmission patterns [7]. This is the first study of its kind in the Republic of Cyprus at a time when there is complete lack of data regarding the sexual patterns and HIV prevalence among Cypriots MSM. Thus, the aim of the study was twofold: a) to estimate the HIV point prevalence and b) to describe risky sexual behaviors among MSM in the Republic of Cyprus.

## Methods

### Study design

The study has been carried out from January 2011 to January 2012. In this descriptive cross-sectional study, time location sampling (TLS) was used to recruit members of high risk groups who are congregated in gay-oriented venues. The aforementioned sampling has been described elsewhere in more detail [13–15]. Briefly after an extensive formative research, the sampling frame was designed, including data on the potential 4 hour adjusted venue-day-time units (VDTUs) of the eligible venues for safety and adequate lighting conditions as well as the size of estimated population frequented in theses venues. Our mapping revealed three categories of MSM venues in the Republic of Cyprus: gay friendly venues, dance clubs and events organized by the community of a Non-Governmental Organization (NGO), in four cities of the Republic of Cyprus (Limassol, Paphos, Larnaca, and Nicosia). Particularly, in the context of the Republic of Cyprus, gay friendly venues in these cities are frequented by MSM and lesbian women. The final sampling frame consisted of 8 venues (approximately 20 VTDUS on a weekly basis). However only one owner has refused to participate. The sampling frame was adjusted to the time period (winter-summer) on a monthly basis. During the second phase VDTUs were selected randomly according to our sampling frame.

The research team (the researcher and two volunteers from an NGO), visited the venue-day-time units. All individuals who entered the venues were informed about the survey and were assessed for eligibility. Eligible participants who had not participated in the study before were invited to participate voluntarily.

### Study population

Eligible participants for the analysis were men who had oral or anal sex with men over the last year, were at least 18 years of age, resided in the Republic of Cyprus for at least the last six months, were able to read and write in either Greek or English and those who agreed to be tested for HIV with an oral test as described further down. Exclusion criteria were the refusal of giving an informed consent and having participated in this study before.

### Procedure and data collection

Participants were asked to complete a self-administered questionnaire in Greek and English. The procedure of filling the questionnaire took about 20 minutes. Answers about sociodemographic characteristics, the frequency of specific sexual behaviors over the last six months, the timing of the last sexual intercourse with a casual or a steady partner, sexual activity and lifestyle factors, HIV testing seeking behaviors and substance use were gathered. Furthermore, self-esteem was measured by using the Rosenberg’s self esteem scale were included.

Before filling the questionnaires, participants who consented to be tested were given an oral HIV test using the OraSure device (ΟraQuick Advance® Rapid HIV- 1/2. OraSure Technologies). The advantages of oral fluid testing in outreach settings for epidemiological and surveillance purposes has been well documented by Mirandola et al. [15]. All participants were given pre- and post test counseling and those with preliminary HIV positive results were referred for further confirmation in a specialized public hospital. Anonymity and confidentiality were guaranteed. Completed questionnaires and oral specimens were linked with the use of an anonymous bar code. Indicators of Global AIDS Response Reporting (GARP) were taken into account (indicator 1.12 that refers to the percentage of men reporting the use of condom the last time they had anal sex with a male partner; indicator 1.13 that refers to the percentage of MSM who received an HIV test in the past 12 months and know their results and indicator 1.14 that refers to the percentage of MSM who are living with HIV) [16]. Prevention leaflets as well as condoms were distributed to all the participants regardless if they agreed to test for HIV.

### Statistical analysis

All of the items were coded and scored and the completed questionnaires were included in the data analysis set. Prevalence of HIV infection (95% confidence interval) was calculated based on positive results of the oral test. Differences between Greek Cypriots and other ethnicity MSM as well as among MSM aged 18-25 and >25 years old were calculated by t-test in the case of continuous variables (e.g. self-esteem) and chi-squared tests in the case of dichotomous variables (e.g. age groups, educational level, alcohol and cocaine use, condom use in the last anal sex, ethnicity, sexual identity, tested for HIV in the last 12 months. Crude and adjusted odds ratios with 95% confidence intervals (CI) for unprotected anal intercourse (UAI) were calculated for each factor in multivariate logistic regression models. P values < 0.05 were considered to be statistically significant. All the analyses were performed in IBM-SPSS-19. Self-esteem was measured by using the Rosenberg self-esteem scale (10-item scale with theoretical range 0-30, with higher values indicating higher self-esteem). Cronbrach’s alpha was calculated.

### Ethical approval

The study was carried out after approval was granted by the Cyprus National Bioethics Committee (EEBK/EP/2010/09).

## Results

### Socio-demographic characteristics

A total of 240 subjects were recruited in the study. The initial sample of 240 individuals consisted of 15 lesbian women and 225 men who met the inclusion criteria. However, 200 men consented to be tested for HIV, (our final sample) demonstrating an overall response rate 89%. The mean age (SD) of the sample was 29 ± 6.6 years old while the majority of MSM (83.9%) were G/C. In terms of sexual self-identification, 74.9% identified themselves as gay whilst 13.3% as heterosexual. The majority of the participants (68.5%) had tertiary level education. Table 1 presents the socio-demographic characteristics of the MSM.

**Table 1:** **Socio-demographic characteristics of MSM in Republic of Cyprus, 2011-2012 (n = 200)** ^**a**^

Variables	Ν	%
Age group		
18-25 years	51	28
>25 years	130	72
Language^b^		
Greek	169	84.5
English	31	15.5
Marital Status		
Single	184	92.0
Married	7	3.5
Divorced	4	2.0
Widowed	1	0.5
In cohabitation	4	2.0
Ethnicity		
Greek Cypriots	168	84.0
Turkish Cypriots	18	9.0
Greek	8	4.0
Other nationalities	6	3.0
Education		
Primary school	6	3.0
Secondary school	57	28.5
College degree	24	12.0
University degree	80	40.0
Master/PhD degree	33	16.5
Occupational Status		
Civil Servant	55	27.8
Private Servant	74	37.4
Worker	12	6.1
Businessman	10	5.0
Unemployment	9	4.5
Self-employment	20	10.1
Student	18	9.1
Sexual identity		
Homosexual/Gay	146	74.9
Bisexual	23	11.8
Heterosexual	26	13.3
Living Arrangements		
With parents	83	41.5
Cohabiting	9	4.5
Alone	102	51.0
Other	6	3.0

MSM: Men who have sex with men.

^a^Note: for some variables totals may not add up due to missing values.

^b^This refers to language of communication with the participants for the purposes of the study.

### HIV prevalence and HIV test seeking behavior

Two hundred participants were tested for HIV. Five respondents were HIV positives indicated a point prevalence of HIV infection of 2.5% (95% CI 0.08% to 5.7%). Two out of the five men who were identified as HIV positives through the use of the rapid test were aware of their HIV status. Almost all the respondents (n = 198) wanted to have access to their oral test results.

Approximately one third of the participants (36%) had not been tested for HIV during the last 12 months. Of those who had been tested, 79.5% had received their result. Regarding their knowledge about HIV testing sites, 69.7% reported they knew where they can get tested for HIV. In the bivariate analysis, men older than 25 years of age were more likely to report that they knew where they can get tested if they wanted to (83.7%), to have been 3tested for HIV in the last year (76.6%) and to have received the results (90.6%) compared to those in the younger age group i.e. 18-25 years old (all p < 0.001).

### Sexual practices

Overall, 121 (60.5%) respondents reported having sexual contacts with women in the last year. The median number of female and male partners in the last year were 4 (IQR: 0-30) and 2 (IQR: 0-50) respectively.

As far as condom use during the last sexual intercourse is concerned, 54 out of 180 MSM reported that they didn’t use condom in their last anal intercourse, indicates an UAI prevalence of 30%. Additionally, 82% (164/200) of the respondents reported having unprotected oral sex. Sixty four point nine percent and 34.5% of the participants reported having had sexual contact with a casual partner and a steady partner respectively the last time they had sexual contact. The proportion of MSM who had unprotected anal intercourse last time and had sex with steady partner was higher (51.9%) compared to the proportion of unprotected anal sex with a casual partner (20.7%).

### Alcohol and illegal drug use

The percentage of the respondents reporting having used alcohol or cocaine between at least a few to many times before or during the sexual contact was 59.5% and 10% respectively while 39.5% and 87.2% reported that they had never used alcohol or cocaine. Alcohol and cocaine use as well as use of lubricants did not differ significantly by age or ethnicity.

### Self esteem

A total of 178 MSM have responded to all items on the Rosenberg’s self-esteem scale. The mean (±SD) score was 12.52 ± 3.8. Internal consistency measured by Cronbrach’s alpha was 0.90, which is considered to be acceptable [17].

### Adjusted and unadjusted odds ratios of factors associated with last unprotected anal intercourse

In the bivariate logistic analysis, alcohol use (OR 2.2, 95% CI 1.09 to 4.44) and cocaine use (OR 6.79, 95% CI 2.8 to 16.5) were associated with decreased odds of condom use during the last anal intercourse. High level of educational attainment are associated with increased odds of condom use during the last intercourse (OR 0.29, 95% CI 0.15 to 0.57). Regarding the type of sexual partners, MSM who had sex with casual partners, were more likely to use condoms in their last anal intercourse compared to those who had anal sex with steady partners (OR 0.25, 95% CI 0.12 to 0.5). Self-esteem was not associated with condom use during the last anal contact in the univariable analysis. When all these variable were entered into a stepwise multivariate regression model adjusting for the effect of each other, the association of alcohol use with unprotected anal sex attenuated (and was no longer statistically significant) whilst a negative association with levels of self-esteem was observed (AOR 0.83 95% CI 0.72-0.96) (see Table 2).

**Table 2:** **Factors associated with last unprotected anal intercourse among MSM in Republic of Cyprus, 2011-2012**

	Condom use in last sexual anal intercourse			
Covariates	n/N	%	Unadjusted odds ratio (95% CI)	Adjusted odds ratio (95% CI)
Sexual Identity				
Homosexual	93/136	68.4	0.95 (0.36-2.47)	
Heterosexual	16/23	69.6	1	
Sexual Identity				
Bisexual	16/20	80.0	1.75 (0.43-7.17)	
Heterosexual	16/23	69.6	1	
Age group				
18-25	37/52	71.2	1.1 (0.54-2.25)	
>25	87/126	69.0	1	
Ethnicity				
Greek Cypriots	107/152	70.4	1.0 (0.41-2.45)	
Other	19/27	70.4	1	
Education				
Elementary	30/58	51.7	0.29 (0.15-0.57)*	0.23 (0.09-0.55)
University degree	96/122	78.7	1	1
Alcohol use				
No	54/69	78.3	2.2 (1.09-4.44)*	
Yes	62/100	62.0	1	
Cocaine use				
No	103/135	76.3	6.79 (2.8-16.5)*	3.78 (1.21-11.83)
Yes	9/28	32.1	1	1
Sexual partner in last sexual intercourse				
Steady partner	27/55	49.1	0.25 (0.12-0.5)*	0.18 (0.08-0.45)
Casual partner	96/121	79.3	1	1
Correct knowledge regarding HIV transmission ways of the transmission^a^				
Yes	97/142	68.3	0.67 (0.29-1.53)	
No	29/38	76.3	1	
Tested for HIV in last 12 months				
No	42/55	76.4	0.98 (0.46-2.18)	
Yes	82/107	76.6	1	
Self-esteem	Mean value		Odds ratio (95% CI)	
	12.55		1.04 (0.95-1.13)	0.83 (0.72-0.96)

MSM; men who have sex with men: CI; confidence interval: HIV; human immunodeficiency virus.

^a^The percentage of respondents who answered correct to all five questions according to GARP indicator.

*Statistically significant.

## Discussion

To the best of our knowledge, this is the first epidemiological bio-behavioral survey among MSM in the Republic of Cyprus. However the findings have to be explained with caution. This study provides useful scientific data on HIV point prevalence as well as on the risky sexual behaviors that contribute to HIV epidemic expansion among MSM. The aforementioned data contribute to the understanding of HIV transmission patterns among Cypriots MSM that are pertinent for the design and implementation of appropriate and targeted interventions.

The prevalence of HIV infection among MSM in the Republic of Cyprus remains at low levels (2.5%) and in line with figures observed in other studies in Central Europe [12, 15]. Almost 88.8% of the respondents agreed with the oral test while 99% of them were willing to have access to the results, demonstrating that Cypriots MSM are willing to participate in such studies. This is an important and encouraging finding as the Republic of Cyprus is a small country in which anonymity plays an important role in seeking an HIV test.

With regard to unprotected anal intercourse, approximately one third of MSM reported that they did not use condom in their last anal sexual intercourse. Similar prevalence rates of unprotected anal intercourse in recent sexual contacts have been documented in similar studies elsewhere [18, 19]. Nevertheless, according to our findings, only 18% (34/200) of the participants reported condom use in their last oral sexual contact. Condom use during oral sex among Cypriots MSM is slightly higher compared to the figures observed in a study performed in six European cities, in which the proportion of consistently condom use in oral sex ranged between 5.8% to 15.1% and it was dramatically lower compared with anal sex [15].

On the other hand, Cypriots MSM seem to use condom with their casual partners more consistently than with their steady partners. Almost three out of four men have used condom in their last intercourse with their casual partners. Unprotected sex with steady partners is common behavior adopted by MSM [20, 21]. The aforementioned sexual behavior was described by Prestage et al. where participants were more likely to have unprotected anal intercourse with sexual partners they knew well [22]. Based on data revealed from the interviews during the formative step, MSM in the Republic of Cyprus use condoms more often with their casual sexual partners because they are not willing to place themselves at risk as well as their steady male or female sexual partners, even if these relationships are not exclusively monogamous.

According to our findings, those older than 25 years of age were more likely to have been tested for HIV and having received their result of an HIV test compared to younger MSM and know where they can be tested if they are willing to do so. The impact of age on HIV testing behaviors has also been described by other researchers [22–25]. The low proportion of MSM aged <25 years old regarding the three aforementioned variables, demonstrate the urgent need of targeted interventions in order to increase awareness of HIV testing sites as well as of the benefits of early diagnosis.

This study also revealed that lower level of education, cocaine use and sexual contact with steady partner are significantly associated with last unprotected anal intercourse. The impact of alcohol and illegal drug use by MSM before or during sexual contact has been demonstrated in other cross-sectional studies [9, 26]. The level of educational background and socioeconomic status have been described as factors that influence the adoption of risky behaviors [27]. Moreover, in the same study [27] higher educational attainment was related to race or ethnicity. Furthermore, in a study in Estonia, alcohol use combined with other factors such as lower education was negatively associated with condom use during the last intercourse [28].

In the current study the self-esteem mean score is considered to be low and was not associated with the last unprotected anal intercourse. According to Moskowitz et al. [11], self-esteem was an independent factor for HIV disclosure but not for condom use. The impact of psychosocial factors has been described also by Folch et al. [9]. Consequently, a more in-depth analysis of the psychosocial factors that contribute to the adoption of risky sexual practices with different types of sexual partners is needed in order to design targeted prevention interventions in MSM.

Some limitations need to be considered. Firstly, de jure TLS methodological approach is a non probabilistic method and consequently it is not feasible to capture all the member of the target population [29]. Particularly, MSM who do not socialize and do not frequent gay friendly venues were not reached as a result of the study design. Another limitation is the refusal of one venue owner to cooperate. However, while not inclusion of some venues might be problematic, it is assumed that due to the small size of the island and the small number of gay venues, MSM tend to frequent at several of these venues, even in cities on the island other than their usual place of residence, thus it is likely that some of the clients of the non-participating site were anyway recruited in the study elsewhere. Finally, it was not feasible to link biological and behavioral data due to limited number of HIV positive results.

## Conclusions

In summary, our findings give a broad first overview regarding the factors that affect sexual behavior and HIV testing seeking behaviors among MSM in the Republic of Cyprus. Policy makers and civil society should cooperate for the implementation of prevention campaigns at community level. Specific actions should promote anonymous and free of charge HIV testing as well as HIV testing sites expansion in broader level among MSM community. Targeted interventions should be implemented focusing in particular to the needs of younger MSM. Finally, the establishment of second generation surveillance system in the Republic of Cyprus could be a scientific tool to design effective interventions as well as to evaluate them.

## References

[1] Centers for Disease Control and Prevention (CDC) (2013). HIV in the United States: At A Glance.

[2] Public Health Agency of Canada (PHAC): HIV and AIDS in Canada: Surveillance Report to December 31st, 2012. Available at http://www.phac-aspc.gc.ca/aids-sida/publication/survreport/2012/dec/index-eng.php

[3] European Centre for Disease Prevention and Control (ECDC)/WHO Regional Office for Europe (WHO) (2013). HIV/AIDS surveillance in Europe 2012. Stockholm: European Centre for Disease Prevention and Control.

[4] Statistical Service of the Republic of Republic of Cyprus (2013). New Publication: Demographic Report 2010-2011.

[5] Republic of Cyprus Ministry of Health (2013). HIV/AIDS National data.

[6] Elford J, Jeannin A, Spencer B, Gervasoni JP, van de Laar MJ, Dubois- Arber F, HIV and STI Behavioural Surveillance Mapping Group: The HIV and STI Behavioural surveillance among men who have sex with men in Europe. Euro Surveill. 2009, 14 (47):10.2807/ese.14.47.19414-en 19941807

[7] Bozicevic I, Voncina L, Zigrovic L, Munz M, Lazarus JV (2009). HIV epidemics among men who have sex with men in central and eastern Europe. Sex Transm Infect.

[8] Beyrer C (2012). Baral SD, vanGriesven F, Goodreau SM, Chariyalertsak S, Wirtz AL, Brookmeyer R; Global epidemiology of HIV infection in men who have sex with men. Lancet.

[9] Folch C, Muňoz R, Zaragoza K, Casabona J: Sexual risk behavior and its determinants among men who have sex with men in Catalonia, Spain. Euro Surveill. 2009, 14 (47):10.2807/ese.14.47.19415-en 19941806

[10] O’Leary A, Wolitski RJ, Remien RH, Woods WJ, Parsons JT, Moss S, Lyles CM (2005). Psychosocial correlates of transmission risk behavior among HIV-seropositive gay and bisexual men. AIDS.

[11] Moskowitz DA, Seal DW (2011). Self-Esteem in HIV –Positive and HIV-Negative Gay and Bisexual Men: Implications for Risk-Taking Behaviors with Casual Sex Partners. AIDS Behav.

[12] McNair LD, Carter JA, Williams MK (1998). Self-esteem, gender, and alcohol use: Relationships with HIV risk perception and behaviors in college students. J Sex Marital Ther.

[13] Karon JM, Wejnert C (2012). Statistical Methods for the Analysis of Time Location Sampling Sampling Data. J Urban Health.

[14] Pylli M, Raftopoulos V (2012). Descriptions of the sampling research methods used for the hard to reach population in the surveillance of HIV infection. Arch Hell Med.

[15] Mirandola M, Toda CF, Krampac I, Nita I, Stanekova D, Stejlikova D, Toskin I, Gios L, Foschia JP, Breveglieri M, Furegato M, Castellani E, Bonavina MG, SIALON network (2009). HIV Bio-behavioural survey among men who have sex with men in Barcelona, Bratislava, Bucharest, Ljubljana, Prague and Verona, 2008-2009. AIDS Behav.

[16] Joint United Nations Programme on HIV/AIDS (UNAIDS) and World Health Organization (WHO (2013). Global AIDS Response Progress Reported 2013 Construction of Core Indicators for monitoring the 2011 UN Political Declaration on HIV/AIDS.

[17] Cronbach LJ (1951). Coefficient alpha and the internal structure of tests. Psychometrika.

[18] Rhodes SD, Yee LJ, Hergenrather KC (2006). A community-based rapid assessment of HIV behavioural risk disparities within a large sample of gay men in southeastern USA: A comparison of African. American. Latino and white men. AIDS Care.

[19] Mansergh G, Naorat S, Jommaroeng R, Jenkins RA, Stall R, Jeeyapant S, Phanuphak P, Tappero JW, van Griensven F (2006). Inconsistent Condom use with steady and Casual Partners and Associated Factors among Sexually-Active Men who Have Sex with Men in Bangkok, Thailand. AIDS Behav.

[20] Folch C, Marks G, Esteve A, Zaragoza K, Muñoz R, Casabona J (2006). Factors associated with unprotected sexual intercourse with steady male, casual male, and female partners among men who have sex with men in Barcelona, Spain. AIDS Educ Prev.

[21] Ramanathan S, Chakrapani V, Ramakrishnan L, Goswami P, Yadav D, Subramanian T, George B, Paranjape R (2013). Consistent condom use with regular, paying, casual partners and associated factors among men who have sex with men in Tamil Nadu, India: findings from an assessment of a large-scale HIV prevention program. BMC Public Health.

[22] Prestage G, Jin F, Grulich A, de Wit J, Zablotska I, George B, Paranjape R (2010). Gay Men are Less likely to Use Condoms with Casual Sex Partners They Knew 'Well’. AIDS Behav.

[23] Sandford TGM, Nel J, Litt et Phil D, Rich E, Reddy V, Yi H (2008). HIV testing and self-reported HIV status in South African MSM: Results from a community-based survey. Sex Transm Infect.

[24] Berghe W, Nöstlinger C, Buvé A, Beelaert G, Fransen G, Fransen K, Laga M: A venue-based HIV prevalence and behavioral study among men who have sex with men in Antwerp and Ghent, Flanders, Belgium, October 2009 to March 2010. Euro Surveill. 2011, 16 (28):21794222

[25] Chow EPF, Jing J, Feng Y, Min D, Zhang J, Wilson DP, Zhang X, Zhang L (2013). Pattern of HIV testing and multiple sexual partnerships among men who have sex with men in China. BMC Infect Dis.

[26] Celentano DD, Valleroy LA, Sifakis F, MacKellar DA, Hylton J, Thiede H, McFarland W, Shehan DA, Stoyanoff SR, LaLota M, Koblin BA, Katz MH, Torian LV, Young Men's Survey Study Group (2006). Associations between substance use and sexual risk among very young men who have sex with men. Sex Transm Dis.

[27] Celentano DD, Sifakis F, Hylton J, Torian LV, Guillin V, Koblin BA (2005). Race/Ethnic Differences in HIV Prevalence and Risks Among Adolescent and Young Adult Men who have sex with men. J Urban Health.

[28] Tripathi A, Rüütel K, Parker RD: HIV risk behavior knowledge, substance use and unprotected sex in men who have sex with men in Tallinn, Estonia. Euro Surveill. 2009, 14 (48):10.2807/ese.14.48.19429-en 20003896

[29] Semaan S, Lauby J, Liebman J (2002). Street and Network Sampling in Evaluation Studies of HIV Risk-Reduction Interventions. AIDS Rev.

[CR30] The pre-publication history for this paper can be accessed here:http://www.biomedcentral.com/1471-2334/14/432/prepub

